# Weakly supervised semantic segmentation for MRI: exploring the advantages and disadvantages of class activation maps for biological image segmentation with soft boundaries

**DOI:** 10.1038/s41598-023-29665-y

**Published:** 2023-02-13

**Authors:** Shaheen Syed, Kathryn E. Anderssen, Svein Kristian Stormo, Mathias Kranz

**Affiliations:** 1 Department of Seafood Industry, Nofima AS, P.O. Box 6122, 9291 Tromsø, Norway; 2 Department of Computer Science, UiT The Arctic University of Norway, Hansine Hansens veg 18, 9009 Tromsø, Norway; 3 PET Imaging Center Tromsø, University Hospital North-Norway (UNN), Hansine Hansens veg 67, 9009 Tromsø, Norway; 4 Nuclear Medicine and Radiation Biology Research Group, UiT The Arctic University of Norway, Hansine Hansens veg 18, 9009 Tromsø, Norway

**Keywords:** Image processing, Machine learning

## Abstract

Fully supervised semantic segmentation models require pixel-level annotations that are costly to obtain. As a remedy, weakly supervised semantic segmentation has been proposed, where image-level labels and class activation maps (CAM) can detect discriminative regions for specific class objects. In this paper, we evaluated several CAM methods applied to different convolutional neural networks (CNN) to highlight tissue damage of cod fillets with soft boundaries in MRI. Our results show that different CAM methods produce very different CAM regions, even when applying them to the same CNN model. CAM methods that claim to highlight more of the class object do not necessarily highlight more damaged regions or originate from the same high discriminatory regions, nor do these damaged regions show high agreement across the different CAM methods. Additionally, CAM methods produce damaged regions that do not align with external reference metrics, and even show correlations contrary to what can be expected.

## Introduction

Deep learning has been a game-changer for the field of computer vision since the breakthrough in 2012 when a deep learning-based model^1^ achieved a significant reduction in error rate on a number of benchmark tests during the ImageNet Large Scale Visual Recognition Challenge (ILSVRC)^2^. Since then, the use of deep learning in computer vision has led to groundbreaking results in image classification, object detection and localization, and semantic segmentation^3–13^.

Semantic segmentation assigns a class label to each pixel in an image, providing information about the location, shape, and size of objects within the image, instance segmentation enables the unique identification of objects belonging to the same class. The main goal of image segmentation is to simplify image representation for easy analysis by machines and to understand the scene. In medicine, it’s applied to a variety of image modalities such as X-ray, visible light imaging, positron emission tomography, computerized tomography, and magnetic resonance imaging (MRI)^14^. Deep learning-based techniques have surpassed traditional image segmentation methods in terms of accuracy and efficiency. Many current semantic segmentation models are based on Fully Convolutional Network (FCN) which uses convolutional neural network (CNN) to learn hierarchies of features. However, creating pixel-level annotation for these models is laborious, time consuming, and expensive.

Weakly Supervised Semantic Segmentation (WSSS) is an alternative solution that utilizes image-level labels and class activation maps (CAM) for semantic segmentation. CAM highlights regions in an image where the deep learning model finds discriminative features, typically through superimposing a heatmap on top of the original image. A typical WSSS pipeline starts with an image classification model to predict the class of an image, then CAMs are used to detect the most discriminative regions, to finally use these regions as seed annotations for regular fully supervised segmentation models such as the FCN, U-NET, and Mask R-CNN^4,9,12^. Today, CAM research is focused on enabling CAM to highlight more of the desired object or unexploited regions in an effort to provide a better overlap and increased precision.

In the biological domain, which includes analyzing medical images to detect and diagnose diseases and studying microscopic images of cells and tissues, image segmentation can be challenging for images with soft boundaries or low contrast. Soft boundaries refer to images where the border between the segmentation classes is not clearly defined, while low contrast refers to images where there is little difference in intensity between the segmentation classes. In these cases, the use of image-level labels and CAM methods can prove a useful method for the purpose of image segmentation. In this paper, we explore the usefulness of WSSS on biological images with difficult to separate class regions. More concretely, we explore several CNN classification models and CAM methods to detect, segment, and quantify damaged tissue regions from $T_{2}$-weighted MRI images taken from Atlantic cod (*Gadus morhua*) fillets in their fresh state and after they underwent different freezing treatments (− 5 °C, − 20 °C, − 40 °C) and were thawed again. Our aim here is to use the image-level label, *fresh* ($y=0$) vs. *frozen and thawed* ($y=1$), to estimate the degree of tissue damage caused by the freezing protocol. The muscle structure transforms (i.e., breaks down) unevenly and at different locations throughout the sample as a result of freezing and thawing. This transformation can be seen as a proxy for damaged tissue, and it is expected that less transformation occurs with lower freezing temperatures. Typically, little transformation occurs when freezing to extremely low temperatures such as − 40 °C. In these cases, samples are visually nearly indistinguishable from their fresh counterparts after thawing.

In more detail, we aim to investigate the following: First, we explore the intersection over union (IoU)—a metric to calculate the degree of overlap—between regions of damaged tissue obtained through our CAM approach to damaged tissue regions found by a supervised classification model from our previous study^15^. Within that study, patch level annotations were utilized to train a CNN model to detect regions of damaged tissue through a sliding window approach^16^. Second, how do CAM highlighted regions of damaged tissue correlate with the amount of liquid loss from the samples. The amount of liquid that escapes the sample is an indicator or metric of the amount of fibers that are damaged, and thus to the degree of damaged tissue. More liquid loss can be associated with more damage. These two aims combined provide insights into the relationship and usefulness of CAM methods compared to other existing methods and quantifiable tissue damage metrics. Third, we explore the IoU agreement between CAM regions across different CAM methods and provide insights into the overlap and stability of CAM methods applied on the same CNN classification architecture. In other words, do different CAM methods highlight similar regions when applied to the same model. Fourth and last, we explore the IoU agreement between CAM regions across different CNN classification models. This is similar to our previous aim, albeit in that we calculate similarities of the same CAM method but applied on different CNN architectures. In essence, how similar are regions of damaged tissue when a different CNN model is utilized. The last two aims inform us about the differences between CAM methods and what the effects of CNN models are on the extracted regions.

## Materials and methods

### Experimental design

The experimental design of this study comprised of the following steps:

1. Train several CNN classification models for binary classification into $y=0$ for the fresh MRI images, and $y=1$ for the MRI images after they were frozen to − 5 °C, − 20 °C, and − 40 °C and were thawed again.
2. Obtain CAM images for the class label $y = 1$. These CAM images can be viewed as heatmaps in the range of [0, 1], with higher values indicating stronger discriminative regions belonging to the $y=1$ class . Several threshold values were used to binarize the heatmaps into segmentation masks: $t_{CAM} = \{0.1, \ldots , 0.9\}$ with values $\ge t_{CAM}$ indicating damaged regions set to 1, and lower values set to 0.
3. CAM regions are compared to supervised classification regions from our previous study^15^.
4. CAM regions are correlated with the amount of drip loss.
5. CAM regions are compared across different CAM methods.
6. CAM regions are compared across different CNN classification models.

### Data samples

Our dataset consisted of 32 samples of cod fillets (146 g ± 19 g) divided into three groups from a total of sixteen Atlantic cod (*Gadus morhua*) fish. The raw materials were provided by Tromsø Aquaculture Research Station, Norway. Each sample was taken from the same loin location and vacuum packed (99%) in plastic pouches. Group 1 consisted of 11 random fillets which were frozen to − 5 °C; Group 2 consisted of 11 random fillets which were frozen in still air to − 20 °C; Group 3 consisted of 10 random fillets which were blast frozen to − 40 °C. After freezing for 5 days, the samples were thawed rapidly in a 4 °C circulating water bath for 2 h.

### MRI acquisition

**Figure 1:**
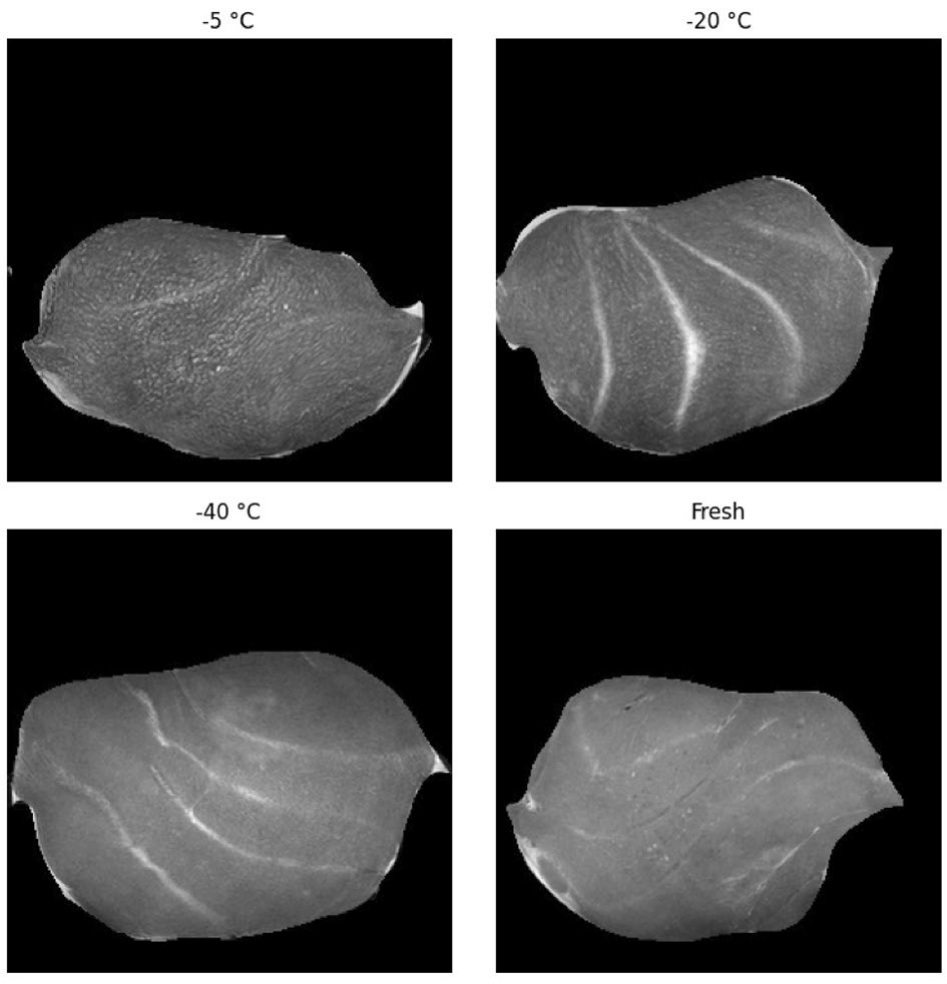
Examples of $T_{2}$-weighted MRI image slices for samples that underwent a − 5 °C, − 20 °C, or − 40 °C freezing and thawing process, as well as a $T_{2}$-weighted MRI image slice in the fresh state (i.e., obtained before freezing and thawing).

MRI images were acquired using a preclinical 7 Tesla MR Scanner (MRS*DRYMAG, MR solutions, Guildford, UK) with a rat quadrature bird cage coil ($\emptyset$ 65 mm, length 70 mm); see supplementary Fig. S1. Images were taken in the axial direction using the Fast Spin Echo $T_{2}$-weighted sequence. Repetition time (TR) was 8s, slice thickness was 1 mm, and the number of slices was 54. Field of View was 60mm and each image was 256 × 256 pixels with 12-bit gray scale values, giving a resolution of approximately 240 μm. Total measuring time per sample was 4 mins. Examples of the $T_{2}$-weighted MRI images for the fresh and frozen/thawed states are shown in Fig. 1. All samples were first scanned in their fresh state and then again after they were frozen and thawed.

### Data pre-processing

MRI images were read from the DICOM format with the python library pydicom (v.1.4.2) and stored in Hierarchical Data Format 5 (HDF5) for offline processing. The background was removed by converting the MRI images to 8 bits, applying a max filter, erosion, and Gaussian blur. These steps were necessary to increase the distinction between the foreground and the background, as the background contained some image artifacts caused by the plastic vacuum packs surrounding the cod fillets. Next, the k-means ($k=2$) clustering algorithm was applied to mask the background from the foreground. Data was randomized and split into a training set (80%), validation set (10%), and test set (10%) with procedures to avoid data leakage commonly present when working with MRI slices^17^. Data augmentation was furthermore applied to the training set by adding images with up to 45 degree rotation, horizontal as well as vertical flipping (samples are invariant to flipping), as well as width and height shift of up to 20%. In addition, contrast adjusted images were added by performing three different contrast enhancement techniques: (i) contrast stretching, (ii) histogram equalization, and (iii) contrast limited adaptive histogram equalization. To enable a supervised classification process, samples were divided into two output classes. The first class ($y=0$) contained the fresh images, and the second class ($y=1$) contained the samples that underwent a freezing and thawing process of − 5 °C, − 20 °C, and − 40 °C.

### Classification models

Several deep learning architectures have been influential in progressing the field of computer vision in that they have become standards and used as building blocks for many other image classification and segmentation architectures. Describing each of them is beyond the scope of this paper, but the interested reader can find succinct descriptions in the available review papers^8,9,14,18^, or by reading the referenced source papers below.

To perform image classification, we used the following six CNN architectures (backbones); in alphabetical order:

1. DenseNet121^19^, significant improvements on CIFAR-10, CIFAR-100, SVHN, and ILSVRC benchmark.
2. ResNet50^20^, $1st$ place in 2015 ILSVRC classification task.
3. Inception V3^21^, new state of the art results on the 2012 ILSVRC benchmark.
4. Inception V4^22^, new state of the art results on the 2012 ILSVRC benchmark.
5. VGG16^23^, $1st$ and $2nd$ place of the 2014 ILSVRC in the image localization and classification tasks.
6. Xception^24^

After the AlexNet architecture won the ILSVRC challenge in 2012^1^, newer architectures tended to go even deeper, with more hidden layers and an increase in the number of parameters. However, in recent years, the focus has been to increase classification performance by reducing the size and number of parameters of the architecture.

All backbone architectures were trained for a total of 2500 epochs with early stopping enabled if the validation error did not increase for 200 epochs. We trained with stochastic gradient descent and a learning rate of 5 × $10^{-3}$. All training was performed on a 3 × GPU RTX Titan with a Xeon W-2295 36-thread CPU on Ubuntu v.18.04 with TensorFlow 2.1^25^. Batch size was 32.

### Class activation maps

With CNNs making better and better predictions on image classification tasks, research has simultaneously focused on the internal mechanisms of CNN and why they make specific prediction. Earlier work has shown that CNNs can perform object detection without having been given labels on the location of the object^26^. This has led to the development of class activation maps as a method to indicate the discriminative image regions used by the CNN to identify that category^27^. In essence, the weights of the final output layer are projected back onto the (last) convolutional feature map to identify the importance of the image regions. This importance is typically displayed with a heatmap superimposed onto the original image. The higher the value of the heatmap, the more discriminative those areas are for classification. These high discriminatory regions show promising results for object localization through bounding boxes, as well as for image segmentation and are called weakly supervised object localization (WSOL) and weakly supervised semantic segmentation (WSSS).

One of the challenges of using CAM for either object localization or segmentation is that CAM focusses only on the most discriminative features, resulting in a sparse and incomplete estimate of the target object^28^ without precise representation of their boundaries^29^. This is a direct result of CAM being optimized for discrimination across class labels rather than for the desired pixel-level estimation task. New lines of research focus on enabling CAM to highlight more of the desired object or unexploited regions in an effort to provide better overlap and increased precision. Several of such methods are proposed, of which we will highlight five of them used in this study.

#### Notation

A CNN model takes as input an $X \in \mathbb {R}^{d}$ and outputs *Y*, which is a probability distribution over the number of classes *c*. The probability of a given class is denoted as $Y^{(c)}$. The activations for the last convolutional layer is denoted as *A*, with the index *k* representing the activation map of the $kth$ channel, which becomes $A_{k}$. Technically, the respective convolutional layer *l* can be part of the notation but this is typically set to the last convolutional layer.

#### Grad-CAM

Gradient-weighted Class Activation Mapping (Grad-CAM)^30^ is a generalization of the CAM^27^ method proposed by Zhou et al. In contrast to CAM, which restricted the CNN architecture to not contain fully connected layers, Grad-CAM can be applied to a broader range of CNN architectures as it can be applied without altering the original CNN architecture or needing to retrain existing models. Grad-CAM is class discriminatory, which enables highlighting one class within an image and excluding other classes, and vice versa. This in contrast to guided back-propagation and deconvolution, where visualizations with respect to different classes are nearly identical. Grad-CAM utilizes the gradient information flowing into the last convolutional layer, right before a fully connected layer (if any), which captures both high-level semantics and detailed spatial information.

Grad-CAM first computes the gradient of the score for a class, $Y^{(c)}$ before applying a softmax, for example c = ‘dog’, with respect to the feature map activations $A_{K}$ of a convolutional layer, typically the last.

1 $$\begin{aligned} \frac{\partial Y^{(c)}}{\partial A_{k}(i, j)} \end{aligned}$$

The gradients are then global-averaged-pooled over the width (*W*), indexed by *i*, and height (*H*), indexed by *j*, to obtain the neuron importance weights $w_{k}^{(c)}$.

2 $$\begin{aligned} w_{k}^{(c)}=\frac{1}{H \cdot W} \sum _{i=1}^{H} \sum _{j=1}^{W} \frac{\partial Y^{(c)}}{\partial A_{k}(i, j)} \end{aligned}$$

The localization map is then computed as:

3 $$\begin{aligned} L_{Grad-CAM}^{(c)}(x, y)=\text {ReLU}\left( \sum _{k} w_{k}^{(c)} A_{k}(x, y)\right) \end{aligned}$$

where $A_{k}(x, y)$ is the activation of node *k* in the target layer of the model at position (*x*, *y*). The RELU activation function is applied to retain only those features that have a positive influence on the class of interest.

#### Grad-CAM++

As a successor to Grad-CAM, Grad-CAM++^31^ was developed. Where Grad-CAM falls short in localizing multiple occurrences of the same class, as well as being less accurate in covering the class region within an image, Grad-CAM++ aims to cover these shortcomings to a greater extent. Grad-CAM++ computes $w_{k}^{(c)}$ as:

4 $$\begin{aligned} w_{k}^{(c)}=\sum _{i=1}^{H} \sum _{j=1}^{W} \alpha _{k}^{(c)}(i, j) \cdot {\text {ReLU}} \left( \frac{\partial Y^{(c)}}{\partial A_{k}(i, j)}\right) \end{aligned}$$

Compared to Grad-CAM, Grad-CAM++ allows the weights $w_{k}^{(c)}$ to be a weighted average of the gradients as opposed to the global average (Eq. 2). The localization map can then be computed as:

5 $$\begin{aligned} L_{Grad-CAM++}^{(c)}(x, y)=\sum _{k} w_{k}^{(c)} A_{k}(x, y) \end{aligned}$$

#### Score-CAM

 Score-CAM^32^ was developed as an alternative to the gradient based methods Grad-CAM and Grad-CAM++. The authors argue that gradients can be noisy and vanish due to saturation caused by the Sigmoid and RELU activation functions. Additionally, linearly weighting activation maps from different channels may not always be adequate since activation maps with high weights do not always contribute more to the output variable. To circumvent this, Score-CAM extracts the importance of activation maps from the contribution of the highlighted input features to the model output, rather than to the local gradient information.

First, activation maps from the last convolutional layer are upsampled to the dimensions of the original image using bilinear-interpolation. These activation maps are then normalized to [0,1] by computing (again we are not denoting the respective layer *l* since this is set to be the last convolutional layer):

6 $$\begin{aligned} A_{k}(i,j)_{norm} = \frac{A_{k} - min(A_{k})}{max(A_{k}) - min(A_{k})} \end{aligned}$$

Next, each normalized activation map is element wise multiplied ($\odot$) by the original image to obtain a masked image $M_{k}$:

7 $$\begin{aligned} M_{K} = A_{k}(i,j)_{norm} \odot X \end{aligned}$$

The coefficient $w_{k}^{(c)}$ is then being defined as:

8 $$\begin{aligned} w_{k}^{(c)}={\text {softmax}}(Y^{(c)}\left( M_{k}\right) ) \end{aligned}$$

A sum of all activations maps is then computed followed by a RELU activation function.

9 $$\begin{aligned} L_{Score-CAM}^{(c)}(x, y)={\text {ReLU}} \left( \sum _{k} w_{k}^{(c)} A_{k}(x, y)\right) \end{aligned}$$

#### Faster score-CAM

 Faster Score-CAM essentially works in the same way as Score-CAM, with the exception in that it limits $A_{K}$ to contain high variance, skipping the activation maps for channels with low variance. Here we have arbitrarily chosen to include only the 10 largest activation maps of a channel *k*.

#### Layer-CAM

 Where the previous CAM methods commonly focus on deriving class activation maps from the final convolutional layer, Layer-CAM^33^ merges activations from all layers. The authors of Layer-CAM argue that the final convolutional layer will contain low spatial resolution, which in turn results in class activiation maps with coarse localization of the object, limiting the detection of finer details of the object. They furthermore argue that other convolutional layers, specifically the earlier layers, can contain larger spatial resolution, in which more fine-grained details can be localized. Combining activiations maps from several layers, hence the name Layer-CAM, can naturally capture the activations maps from shallow and deeper layers.

Calculation of the activation map for a single convolutional layer is, in essence, similar to that from Grad-CAM, albeit that Grad-CAM assign a global weight $w_{k}^{(c)}$ to the $k_{th}$ feature map $A_{k}$ whereas Layer-CAM uses the variance. For shallower layers, the global weight is inappropriate since the feature maps will capture noisy regions in the background. Layer-CAM thus calculates the weights of location (*x*, *y*) as:

10 $$\begin{aligned} w_{k}^{(c)}={\text {ReLU}}\left( g_{k}^{(c)} \right) \end{aligned}$$

with $g_{k}^{(c)}$ now being the variance of each (*x*, *y*) location calculated as the difference of the gradient to the average gradient. The class activiation map is then calculated by multiplying $w_{k}^{(c)}$ with the feature map.

11 $$\begin{aligned} \hat{A}_{k}(x,y)= w_{k}^{(c)} A_{k}(x,y) \end{aligned}$$

Next, the $\hat{A}_{k}$ are linearly combined along the different *k* channels.

12 $$\begin{aligned} M^{(c)} = \text {ReLU} \left( \sum _{k} \hat{A}_{k} \right) \end{aligned}$$

Finally, to combine the class activation maps from several layers, the layers are scaled with a scale factor $\gamma$ since the activation values from shallower layers are lower. The authors report that a scaling factor of $\gamma = 2$ works best.

13 $$\begin{aligned} L_{Layer-CAM}^{(c)} = \hat{M}^{(c)}=\tanh \left( \frac{\gamma * M^{(c)}}{\max \left( M^{(c)}\right) }\right) \end{aligned}$$

#### CAM post-processing

CAM images were obtained from the last (i.e., deepest) convolutional layer, except for Layer-CAM, which combined multiple layers, and only for $Y^{(c)} = 1 = damaged$. This resulted in CAM images (displayed as heatmaps) in the range of [0, 1] for damaged regions. Note that CAM images in the fresh class were ignored, that is $Y^{(c)} = 0$. Additionally, CAMs were binarized—segmenting damaged from background—for several threshold values $t_{CAM} = \{0.1, \dots , 0.9\}$ with values $> t_{CAM}$ indicating damaged regions set to 1, and lower values set to 0.

### IoU between CAM and supervised classification

CAM images were compared to supervised classification images and the intersection over union (IoU) was calculated between CAM image *A*, and supervised classification image *B* as:

14 $$\begin{aligned} \text {IoU}(A,B) = \frac{| A \cap B |}{| A \cup B |} = \frac{| A \cup B |}{| A | + | B | - | A \cap B |} \end{aligned}$$

where *A* represents a binarized CAM image (e.g., $t_{CAM} = 0.5$), and *B* represents a supervised classification image. Both *A* and *B* have equal dimensions $A_{(H,W)} = B_{(H,W)}$ and represent the exact same sample and the exact same MRI slice. Additionally, *A* and *B* contain 0’s and 1’s, with 1’s representing the damaged regions. Mean IoU (*mIoU*) would then be the average IoU of all MRI slices $i \in I$ of image *A* compared to image *B*.

15 $$\begin{aligned} mIoU = \frac{\sum _{i} \left( \text {IoU}(A_{i}, B_i{})\right) }{|I|} \end{aligned}$$

### Liquid loss

Liquid loss was calculated right after MRI imaging by opening the vacuum sealed bags and collecting the expelled liquid. Liquid loss (LL, %) was determined according to the formula:

16 $$\begin{aligned} LL (\%) = \frac{m_0 - m_L}{m_0} \cdot 100 \end{aligned}$$

where $m_{0}$ is the initial weight of the loin, and $m_{L}$ is the weight of the loin after packaging, first MRI imaging, frozen storage, thawing, and finally the second MRI imaging.

### Agreement across CAM methods

The agreement across CAM methods reflects the degree to which CAM activations obtained from different CAM methods agree with each other while fixing the CNN model. This agreement is measured by calculating the average intersection over union of the unique combinations of CAM methods. Let $c \in C$ be the set of CAM methods with $C = \{ \text {GradCAM}, \ldots , \text {LayerCAM} \}$. Let $m \in M$ be the set of CNN backbone models with $M = \{ \text {DENSENET121},..., \text {XCEPTION} \}$. We calculate the average agreement $\phi$ across a CNN model *m* as:

17 $$\begin{aligned} \phi _{m} =\frac{1}{{| C | \atopwithdelims ()2}} \cdot \sum _{\begin{array}{c} i = 1 \\ i \ne j \\ i<j \end{array}}^{| C |} \sum _{j = 1}^{| C |} \text {IoU} \left( \left( C_{i},m \right) , \left( C_{j},m \right) \right) \end{aligned}$$

where *m* is kept fixed and $\phi _{m}$ represents the agreement of a single MRI slice for model *m* with fixed $t_{CAM}$. We will report on the distribution of all the slices (n=54) and all the samples (n=32), while reporting on results for different $t_{CAM}$ threshold values. Values close to 1, that is $\phi _{m} \approx 1$, mean that CAM regions obtained from different CAM methods are very similar.

### Agreement across CNN models

Similarly to agreements across CAM methods, we calculate the agreement across different CNN models while fixing the CAM method. Again, this agreement is measured by calculating the average intersection over union, albeit now between unique combinations of CNN models. The agreement then becomes:

18 $$\begin{aligned} \phi _{c} = \frac{1}{{| M | \atopwithdelims ()2}} \cdot \sum _{\begin{array}{c} i = 1 \\ i \ne j \\ i<j \end{array}}^{| M |} \sum _{j = 1}^{| M |} \text {IoU} \left( \left( c, M_{i} \right) , \left( c, M_{j} \right) \right) \end{aligned}$$

 Here *c* is kept fixed and $\phi _{c}$ represents the agreement of a single MRI slice for CAM method *c*, for example *c*=GradCam++. Values close to 1 represent high agreement and thus high overlap between CAM regions obtained from different CNN models for the same CAM method.

## Results

### Classification results

A total of six CNN models were trained for binary classification of the MRI image slices. Table 1 shows the accuracy classification performance for the training (80%), validation (10%), and test (10%) datasets. Performance results for each epoch can be found in Fig. S4 the supplementary material. All models show perfect or near-perfect classification performance on training, validation, and test set. These CNN backbone models form the basis for obtaining class activation maps for six CAM methods.

**Table 1:** Classification accuracy of CNN backbones.

CNN backbone	Train (80%)	Validation (10%)	Test (10%)
DENSENET 121	1.0 (± 0.0)	1.0 (± 0.0)	1.0 (± 0.0)
INCEPTION V3	1.0 (± 0.0)	1.0 (± 0.0)	1.0 (± 0.0)
INCEPTION V4	1.0 (± 0.0)	1.0 (± 0.0)	1.0 (± 0.0)
RESNET50	1.0 (± 0.0)	1.0 (± 0.0)	0.99 (± 0.01)
VGG16	1.0 (± 0.0)	1.0 (± 0.0)	1.0 (± 0.0)
XCEPTION	0.99 (± 0.0)	1.0 (± 0.0)	0.99 (± 0.01)

95% confidence intervals between parenthesis. Number of training epochs is shown between parentheses of the CNN backbone column.

### CAM regions

**Figure 2:**
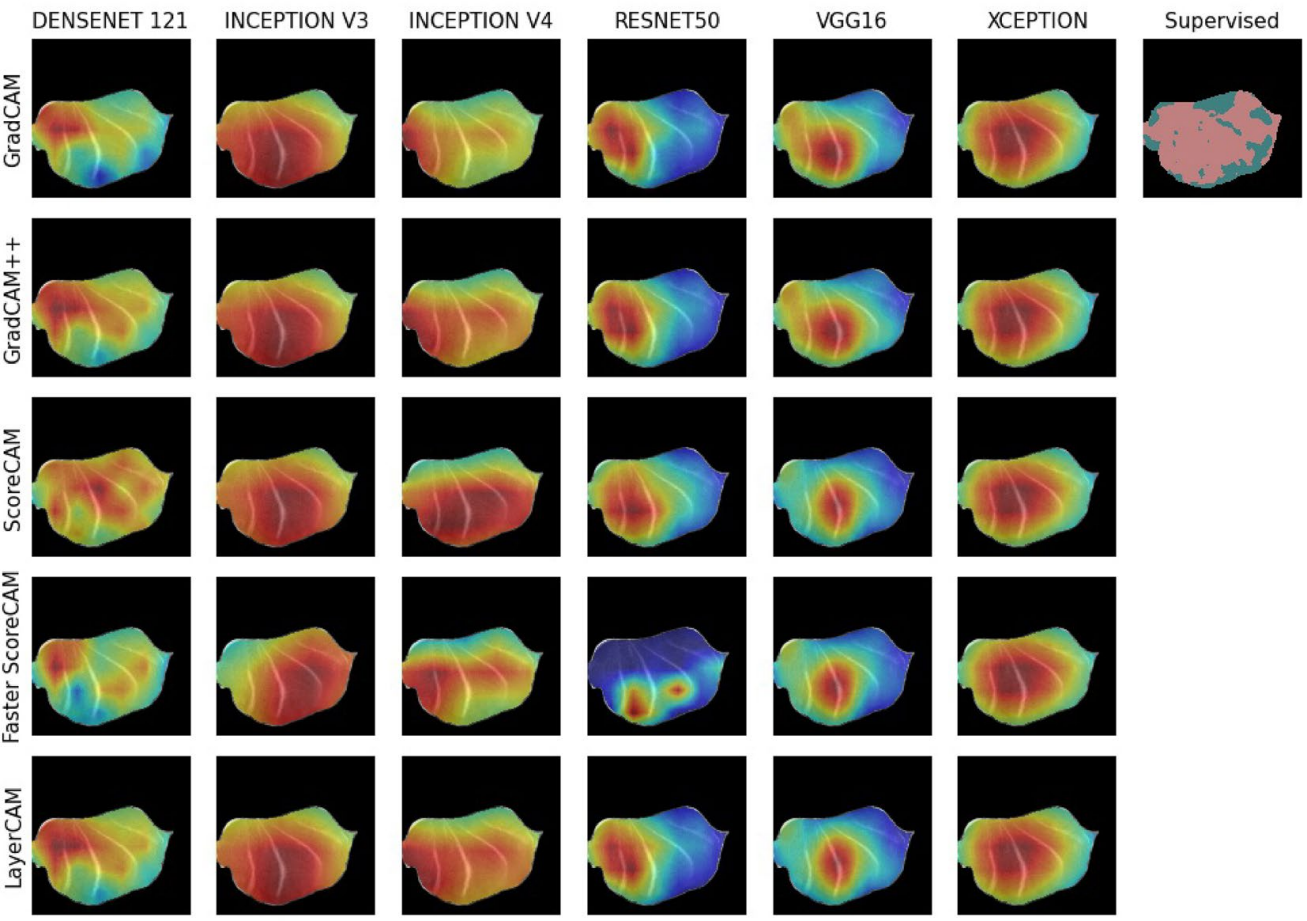
Overview of damaged tissue (y = 1) of different CAM methods and CNN backbone combinations on the same sample. The plot on the top right shows the supervised classification in which the dark pink illustrates the damaged regions.

A random example overview of obtained class activation maps for the damaged class (y = 1) can be viewed in Fig. 2, as well as in Figs. S2 and S3 in the Supplementary Information. Clear differences can be detected among different CNN models, as well as different CAM methods. For example, the resnet50 and vgg16 models show much smaller activated regions across the different CAM methods. In contrast, the inception v3, inception v4, and the xception models show much larger activated regions. In addition, Fig. 2 indicates in the top right corner the damaged regions (in pink) obtained through a supervised cnn model^15^. The differences in activated regions across CAM methods and across CNN models will be presented in the following sections, as well as the level of agreement with damaged regions with supervised classification and the correlation with liquid loss.

### IoU with supervised classification

**Figure 3:**
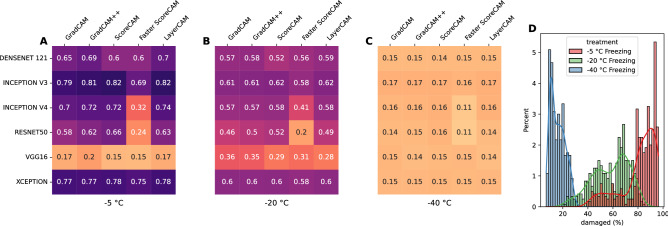
(**A**–**C**) Mean intersection over union (mIoU) between CAM images ($t_{CAM} = 0.5$) and supervised classification separated by the three freezing protocols: (**A**) − 5 °C, (**B**) − 20 °C, (**C**) − 40 °C. (**D**) Shows the distribution of damaged tissue obtained through a supervised CNN model.

The supervised classification of damaged tissues are compared with obtained class activation maps through the mean intersection over union (mIoU) metric, as outlined in “IoU between CAM and supervised classification” section. Figure 3 shows the mIoU for the class activation maps thresholded at $t_{CAM} = 0.5$ for the damaged regions compared to those obtained through a supervised learning process. Values for other threshold values, $t_{CAM} = \{0.1, \ldots , 0.9\}$ can be found in Fig. S5 in the Supplementary Material. It can generally be assumed that freezing at higher temperatures (low freezing rate) is associated with more damage and that damaged regions may represent almost the whole sample. As can be seen from Fig. 3, generally high mIoU values are calculated for samples that have been frozen to − 5 °C, an indication that these damaged CAM regions are consistent with supervised classification regions. The exception here is the vgg16 model, which show much lower values across all CAM methods. Despite high mIoU values for the − 5 °C samples, mIoU values for the − 20 °C are noticeable lower, although most are still above the 0.5 lower bound of what would be an acceptable minimum. This trend continues to even lower mIoU values for the − 40 °C samples. Figure 3D additionally shows the distribution of damaged tissue obtained by a supervised CNN model. Again, this is in line with current understanding of how freezing impacts muscle samples, where slow freezing (at higher temperatures) produces a high degree damage, whereas fast freezing (at lower temperatures) results in much less overall damage.

### Correlation with liquid loss

**Figure 4:**
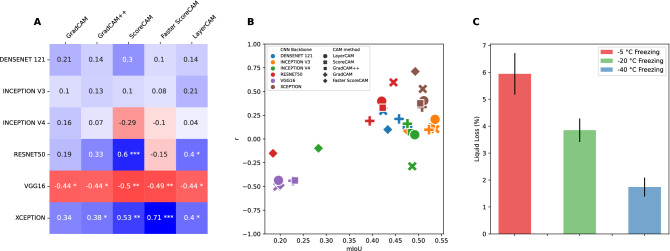
(**A**) Spearman’s rank correlation between damaged CAM regions and the amount of liquid loss for reach CNN model and CAM method combination. (**B**) Overview of the relationship between mIoU scores (x-axis) and the correlation with liquid loss (y-axis) for each CNN model and CAM method combination. (**C**) Liquid loss as a percentage of the weight of the sample for each of the three freezing protocols. Error bars represent the 95% confidence interval. *p < 0.05, **p < 0.01, ***p < 0.001.

As liquid loss can be seen as an indicator for the degree of damage that has occurred within the sample, one would expect the CAM regions to correlate positively. In other words, more damage is typically associated with a higher degree of liquid expelling out of the tissue. Figure 4A displays the Spearman’s rank^34^ correlation with the amount of liquid loss; Pearson correlation could not be performed since normal distribution of the data cannot be assumed, Shapiro-Wilk test $p > 0.05$^35^. Additionally, Fig. 4C shows the percentage of liquid loss for the samples, with a clear decrease in the amount of liquid loss when samples are frozen to lower temperatures.

As can be seen from Fig. 4A, varying degrees of correlations are calculated, both positive as well as negative correlations, with the highest correlation ($r=0.71; \; p<0.001$) for the xception model with the faster score-CAM method. The vgg16 model continues to perform poorly on the correlation results with negative correlations calculated across all CAM methods. Figure 4B shows how the correlation and mIoU values relate to one another. Here the mIoU scores are averaged across the different freezing protocols. Some degree of grouping can be observed, with the vgg16 models performing poorly both on the correlation and mIoU values. In contrast, the inception v3 model performs best with the highest mIoU scores in combination with almost all of the CAM methods. The xception model shows high mIoU values with high correlation scores as well.

### Agreement across CAM methods

**Figure 5:**
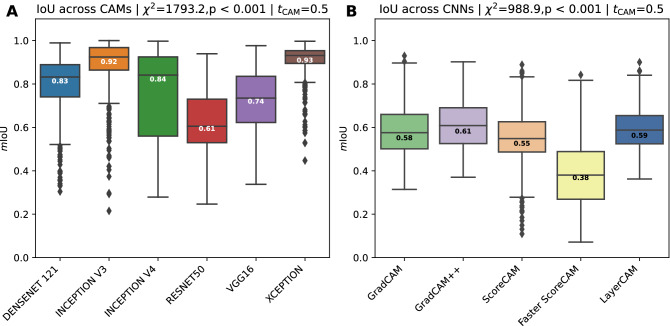
(**A**) The IoU agreement across CAM methods. It reflects the degree to which CAM activations obtained from different CAM methods have a high IoU with each other while fixing the CNN model. (**B**) The IoU agreement across different CNN models while fixing the CAM method. This agreement is measured by calculating the average intersection over union between unique combinations of CNN models. Median values are shown in the boxes.

Agreements across different CAM methods are shown in Fig. 5A. The agreement is essentially comparing the binarized CAM images ($t_{CAM} = 0.5$) for damaged tissue of different CAM methods applied on the same CNN model; see Supplementary Fig. S7 for other $t_{CAM}$ threshold values. In other words, how similar are CAM images obtained from different CAM methods when obtained from the same CNN model, similar to comparing the CAM images column-wise as shown in Fig. 2.

CAM methods applied on the xception model obtain the highest agreement with a median value of 0.93, although some visible outliers are present with much lower scores. An almost equally high agreement score is obtained from the CAM methods applied on the inception v3 model, with a median score of 0.92, although the minimum (i.e., lower whisker) stretches more than that from the xception model. Median values for the densenet121 (0.83) and inception v4 (0.84) model are very similar, albeit that the inception v4 model has a more stretched out interquartile (IQT) range. CAM images obtained from the resnet50 model are the lowest, with a median score of 0.61. A Kruskal-Wallis H test showed that there was a statistically significant difference in IoU scores across the CAM methods, $\chi ^{2}=1793.2, p<0.001$. Similar patterns across the CAM methods with different $t_{CAM}$ threshold values can be observed, where $t_{CAM} \ge 0.5$ causes the IQR to be more stretched and the median value gradually decreases, while maintaining similar differences among the models (Supplementary Fig. S7).

### Agreements across CNN models

Agreements across different CNN models are shown in Fig. 5B. Fig. 5B compares the binarized ($t_{CAM} = 0.5$) CAM images of the same CAM method, but applied on different CNN models. This is similar to comparing the CAM images row-wise from Fig. 2. Other $t_{CAM}$ threshold values can be found in the Supplementary Fig. S8.

Lower mIoU values across the CNN models are obtained due to their individual architectural differences. As can be seen from Fig. 5B, most models obtain similar agreement values, ranging between median scores of 0.55 and 0.61, with the Grad-CAM++ method achieving the highest agreement. A clear outlier is the Faster Score-CAM method, with a median value of 0.38. This is also noticeable when visually analyzing the Faster Score-CAM row in Fig. 2, with different regions highlighted across the various CNN models. A Kruskal-Wallis H test showed that there was a statistically significant difference in IoU scores across the CNN models, $\chi ^{2}=988.9, \; p<0.001$.

## Discussion

CNN classification models are able to detect regions of pixels within an image that show high discriminative power between the different classes. That is, the features that make up those regions are combinations of pixels that are present within one class, and typically not present within the other class or classes. In this paper, we have attempted to detect class specific regions ($y=1$) of pixels that are semantically labeled as damaged regions through freezing and thawing. Here, damaged is used as a proxy for pixel regions dissimilar from images of a fresh state ($y=0$), and encompass the transformation of tissue (i.e., pixels) after freezing and thawing. Through class activation maps, discriminative regions have been made explicit and are quantified by a value between 0 and 1 on a per-pixel basis. Throughout this paper, we have generally set a cut-off threshold of $\ge$0.5 for pixels belonging to the damaged class, with these pixel regions resembling regions dissimilar from those in the fresh state.

### CAM regions

While different CAM methods are unified in purpose, their output can be divergent and non-overlapping^36^. This is visually pertinent in Fig. 2, with different CAM images (i.e., heatmaps) produced across CAM methods and CNN models. Looking at the CAM images obtained from the same CNN model, we see that various CAM methods applied on the resnet50 model produce very different CAM images. Although Fig. 2 only shows CAM images for a single MRI image, similar patterns can be observed by looking at Fig. 5A for the whole dataset. In other words, applying different CAM methods on the same CNN model can produce very different CAM heatmaps and have very low agreement (i.e., low IoU across CAMs). This is to be expected to some degree, as newer CAM methods claim to outperform older CAM methods by highlighting more of the class object. For example, a visual comparison of different CAM methods will typically show the different CAM images obtained from the same sample image^31,33^. The target class object within these images commonly have hard boundaries, e.g. a bird in the sky, and enhanced CAM methods will then highlight more of that object, with the ultimate aim of highlighting the whole object. For example, one CAM method might only highlight the head of the bird, whereas successor (i.e., newer) CAM methods might include, besides the head, also the wings and the legs. For CAM to work better with WSSS, current research is directed towards CAM methods highlighting more of the class object^28,29^, but highlighted areas will originate from the same (e.g., the head of the bird) high discriminative feature region^33^. Given our visual results, see Fig. 2, as well as Figs. S2 and S3, this is not always the case, even though the same CNN model was used. Moreover, different CAM methods can produce different CAM heatmaps, given that they use the same underlying CNN classification model (e.g., architecture, weights, filters, and feature maps), and that these differences do not always originate from the same high discriminatory region. This is surprising since newer CAM methods, typically successor CAM methods which are based of CAM^27^ or Grad-CAM^30^, claim superior results in terms of highlighting more of the class object. This superiority is typically proven through a higher IoU with ground truth labels from the Pascal Visual Object Classes (VOC) dataset^37^.

Although CAM is visually appealing^38^, it is oftentimes difficult to evaluate their reliability because of a lack of ground truth data^36^. We have attempted to use domain knowledge insights—what CAM regions do we expect given the freezing and thawing experiment—as well as correlating and associating with other types of tissue damage quantification metrics. In light of this, the xception CNN model provided CAM regions most in line with our expectations, regardless the used CAM method, see Fig. 2. Inarguably, a varying degree of structural damage is to be expected in a cross-section of the sample. This is because some regions, particularly the center of the sample, experience long periods of phase transitions because of lingering energy. Energy travels slowly due to low thermal conductivity of unfrozen muscle. Additional delay is brought forth as energy, on its path out of the sample, gets entangled in multiple phase transitions. This in turn leads to more pronounced damage from ice crystals as they recrystallize and grow over time^39^. Even if most of the CAM methods predict a non-uniform distribution of damage (Fig. 2), the CAM images obtained from the xception model show heaviest damage in the center of the sample and gradually less toward the edge. It is reasonable to assume that during the freezing process only the edge of the sample, because it is in contact with the freezing medium, experiences sufficient low temperature to stabilize its ice crystals and thereby preventing recrystallization and crystal growth. The damage to the rest of the sample, however, is linked to the duration of phase transitions (and recrystallizations) in the temperature range just barely above freezing point, and this is perceptively illustrated in the CAM images obtained from the xception model. Looking at Fig. 5A, the xception model also shows the highest agreement across the different CAM methods. This agreement is an indication of different CAM methods uncovering similar regions, and that those regions show high overlap between them. This also raises the question whether CAM methods need to be optimized to improve WSSS to enable a higher overlap of the object or regions within an image, or that selecting and optimizing the classification model is a better candidate.

### Comparison to supervised classification and liquid loss

Given that we have empirically demonstrated that different CAM methods and CNN models can produce very different CAM heatmaps, at this stage it is still difficult to say something meaningful about their reliability. To enable some form of real-life reliability, we have compared the damaged CAM heatmaps to results from a supervised classification model^15^, as well as the amount of liquid loss from the samples.

Liquid loss can be seen as an indicator for the degree of damage that has occurred within the sample. This is because the main mechanism of quality deterioration for frozen fish is related to the nature of ice crystals. Formation and recrystallization through phase transitions (freezing/thawing) lead to the formation of large ice crystals, which in turn leads to cell rupture and subsequent liquid loss. In other words, more damaged is typically associated with a higher degree of liquid expelling out of the tissue. For once frozen samples, there is strong evidence that fast processes of freezing and thawing reduce the subsequent liquid loss and that the freezing process is the most critical of the two^40^. This is why different freezing rates, through the different freezing temperatures, are used in this study. Given this knowledge, a high positive correlation should be expected and this is contrary to some CAM methods and CNN models. Although we obtained significant positive correlations for some CAM methods and CNN models (Fig. 4A), most notably the xception model with the Faster Score-CAM method ($r = 0.71, \; p < 0.001$), these then do not reflect the highest IoU overlap with the supervised model (see Figs. 3 and 4B). In contrast, high IoU overlap values are obtained for mainly the Inception v3 model with all CAM methods with the exception of the faster Score-CAM method, with the downside that these then do not produce the desired significant positive correlation with the liquid loss.

The supervised model that we refer to is based on manually labeling areas as damaged and non-damaged through visual inspection^15^. These areas are then transformed into 8 x 8 pixel patches to subsequently classify the whole image through a sliding window approach. One difficulty here is the visual annotation of damaged and non-damaged regions, which can be a very subjective task. Although a clear difference between the fresh state and warmer freezing procedure (e.g. − 5 °C) images can be observed, see for example Fig. 1, this difference becomes less clear for colder freezing procedures (e.g. − 40 °C). More concretely, as can be seen from Fig. 1, the − 5 °C MRI sample image seems wrinkly and can clearly be distinguished from the fresh MRI sample image. This difference becomes less obvious for the − 20 °C image, and visually nearly impossible to detect for the − 40 °C image, even for an experienced MRI physicist. The fact that regions of damaged tissue have soft boundaries makes it even more challenging to separate damaged from non-damaged. Additionally, MRI images are based on 12-bit gray scale values, which can have $2^{12}$ different intensity values. Since all CNN models can almost perfectly classify fresh MRI images from the frozen/thawed images, see Table 1, small variations in pixel intensity values that are hard to detect visually are mathematically easier to exploit. The low IoU overlap, especially for the − 20 °C and even more so for the − 40 °C images (see Fig. 3), can therefore be explained by the shortcomings of visual labeling, and the possibility of CAM images picking up on tiny deviations that are visually the same, but not necessarily seen as damaged.

### Limitations

The work presented here is limited in that it is restricted to one tissue type, and this is likely to affect the generalizability of our results. However, given the tissue involved, we have utilized data augmentation to capture multiple degrees of gray scale variations through three different contrast enhancement techniques: (i) contrast stretching, (ii) histogram equalization, and (iii) contrast limited adaptive histogram equalization. This will likely capture MRI acquisition variations due to the available parameters involved, such as changes to various wait times, pulse lengths, or gradients strengths which can alter the relative intensity of different constituents in the sample.

Additionally, there is currently a plethora of class activation methods available, of which we have explored some of the most utilized ones. However, several others exist that can add additional insights into the findings we present here. These CAM methods are, but not limited to, Smooth Grad-CAM++^41^, Smoothed Score-CAM^42^, Integrated Score-CAM^43^, and Axiom-based Grad-CAM^44^. Typically, all of them will prove some level of increased localization ability in comparison to predecessor CAM methods. Again, improved performance typically relates to images with clear and hard boundaries, and how they perform on soft boundaries image objects is an interesting avenue to explore.

A typical WSSS pipeline will subsequently utilize the CAM images as seed regions for a fully supervised segmentation model (e.g., FCN, U-Net, Mask R-CNN). However, we have not performed this final step as our main goal was to gain insights into the different CAM regions in isolation, without adding another layer of variability that arises when utilizing one or several fully supervised segmentation models.

In conclusion, future research in this area should focus on expanding the scope of the study to include multiple tissue types to increase the generalizability of the results. Another avenue for future research would be to integrate the CAM images as seed regions for a fully supervised segmentation model. This would allow for a more complete evaluation of the performance of the CAM methods and provide insights into the relationship between the CAM regions and the final segmentation results.

## Conclusion

Class activation map (CAM) methods are techniques to gain insights into the most discriminative regions of a class object within an image. Newer CAM methods typically outperform predecessor CAM methods by claiming to highlight more of the class object. For images with clear and hard boundaries, e.g., an image of a bird in the sky, assessing how much of the class object (e.g., the bird) is highlighted is generally a straightforward task. However, for class objects or regions with non-clear or soft boundaries (e.g., within biological images of tissue) this task becomes much harder. We have empirically evaluated several CAM methods applied on several convolutional neural networks (CNN) with the aim of detecting regions of damaged tissue in MRI images of cod tissue. Our results show that CAM methods applied on the same CNN model can produce very distinct regions of damaged tissue, that may not even originate from the same high discrimative region. Additionally, an evaluation of damaged CAM regions with other metrics to quantify tissue damage (e.g., liquid loss, or a supervised classification model), show results contrary to what can be expected. This raises the question that, although visually appealing, how useful CAM methods are when utilized for the purpose of weakly supervised semantic segmentation.

## Supplementary Information

Supplementary Figures.

## Data Availability

The datasets used and/or analysed during the current study available from the corresponding author on reasonable request.

## Code availability

All the Python code that supports the findings of this study are available at https://github.com/Nofima-MRI/cod-class-activations.
