# Supplementary material for: Weakly supervised semantic segmentation for MRI: exploring the advantages and disadvantages of class activation maps for biological image segmentation with soft boundaries
Source: Sci Rep. 2023 Feb 13;13:2574. doi: 10.1038/s41598-023-29665-y (PMC9925800; doi:10.1038/s41598-023-29665-y)
Supplement: Supplementary file 1 — Supplementary Figures. [file 41598_2023_29665_MOESM1_ESM.pdf]

Supplementary Information

Weakly supervised semantic segmentation for MRI: Exploring the advantages and disadvantages of class activation maps for biological image segmentation with soft boundaries

Shaheen Syed[1,2,*], Kathryn E. Anderssen[1], Svein Kristian Stormo[1], and Mathias Kranz[3,4]

[1]Department of Seafood Industry, Nofima AS, P.O. Box 6122, Tromsø, 9291, Norway
[2]Department of Computer Science, The Arctic University of Norway, UiT, Hansine Hansens veg 18, Tromsø, 9009, Norway
[3]PET Imaging Center Tromsø, University Hospital North-Norway (UNN), Hansine Hansens veg 67, Tromsø, 9009, Norway
[4]Nuclear Medicine and Radiation Biology Research Group, The Arctic University of Norway, UiT, Hansine Hansens veg 18, Tromsø, 9009, Norway
[*]corresponding author: shaheen.syed@uit.no

January 19, 2023

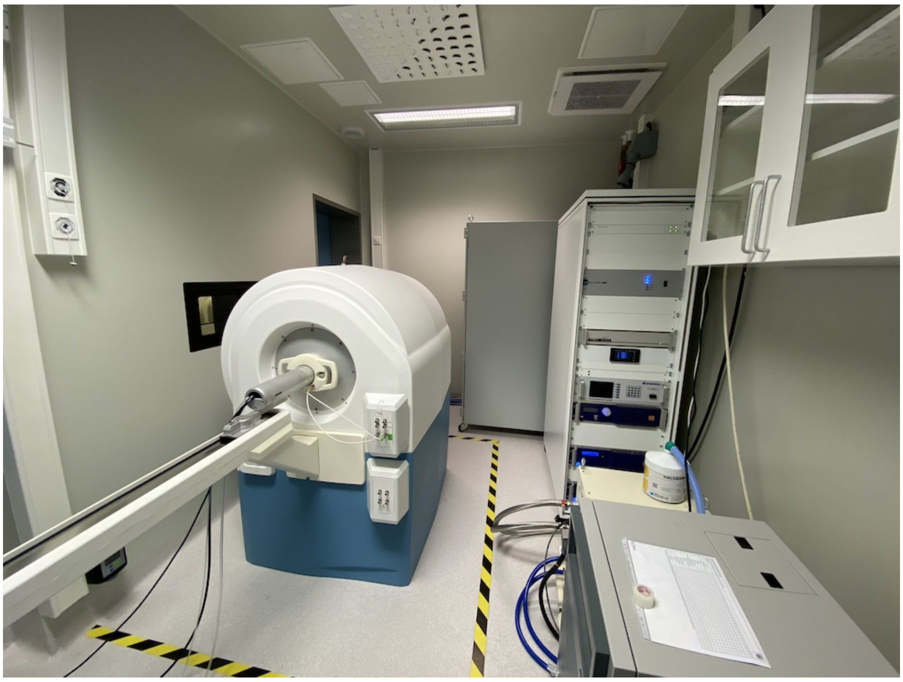

Figure S1: Preclinical 7 Tesla MR Scanner (MRS*DRYMAG, MR solutions, Guildford, UK) used for MRI image acquisition.

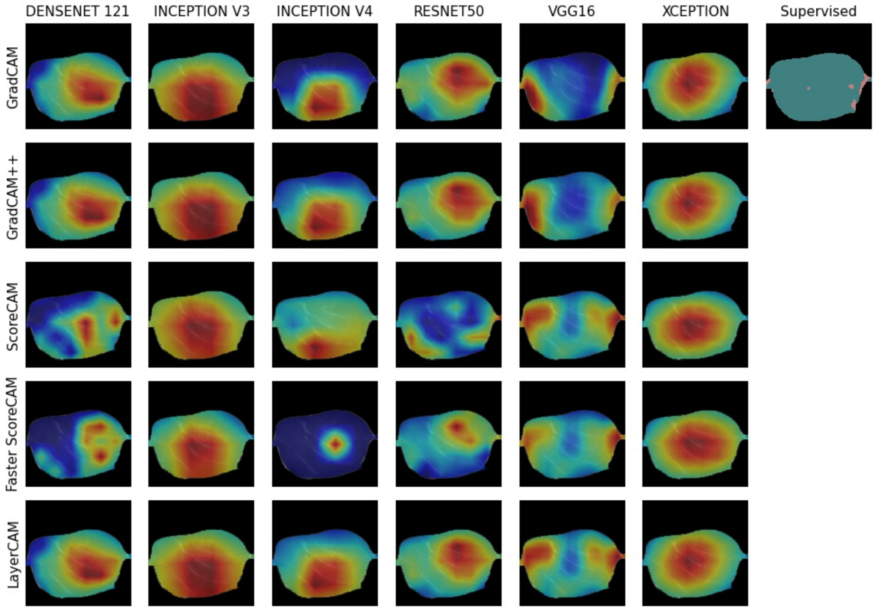

Figure S2: Overview of damaged tissue (y = 1) of different CAM methods and CNN backbone combinations on the same sample. The plot on the top right shows the supervised classification in which the dark pink illustrates the damaged regions.

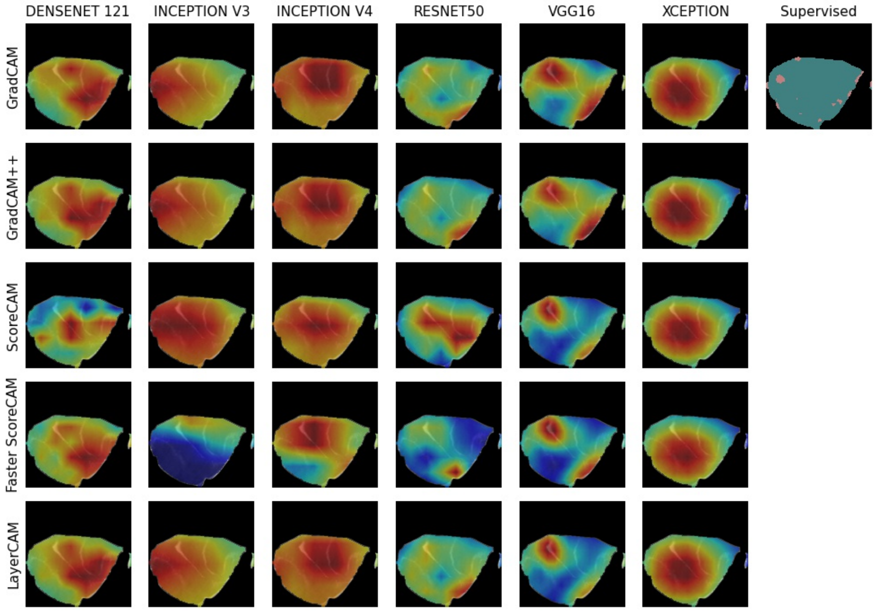

Figure S3: Overview of damaged tissue (y = 1) of different CAM methods and CNN backbone combinations on the same sample. The plot on the top right shows the supervised classification in which the dark pink illustrates the damaged regions.

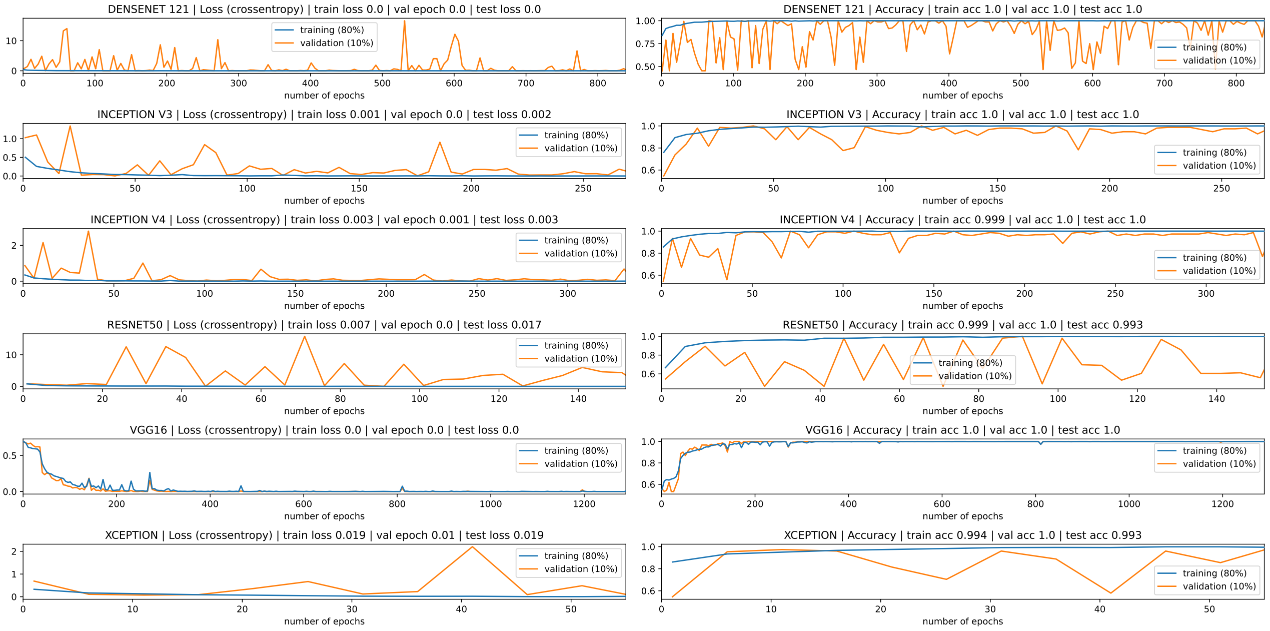

Figure S4: Accuracy and loss (columns) for each epoch (x-axis) and for each of the CNN backbones (rows). Training was done for a maximum of 2,500 epochs with early stopping enabled if the validation error did not increase for 200 epochs. We trained with stochastic gradient descent and a learning rate of 5 x 10^{-3}. All training was performed on a 3 x GPU RTX Titan with a Xeon W-2295 36-thread CPU on Ubuntu v.18.04 with TensorFlow 2.1. Batch size was 32.

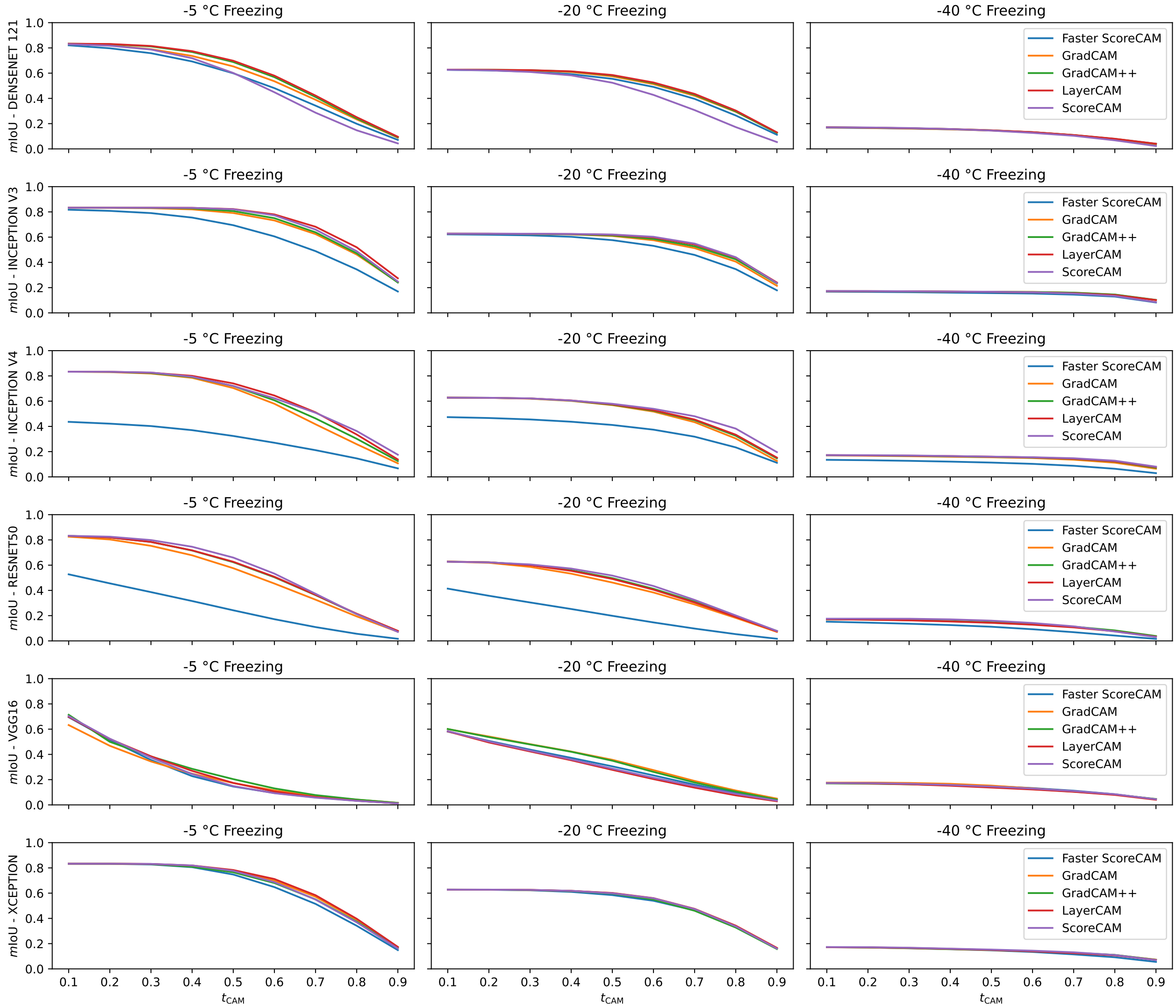

Figure S5: Mean intersection over union (mIoU) between CAM images and supervised classification separated by the three freezing protocols and for different threshold values (t_{CAM}).

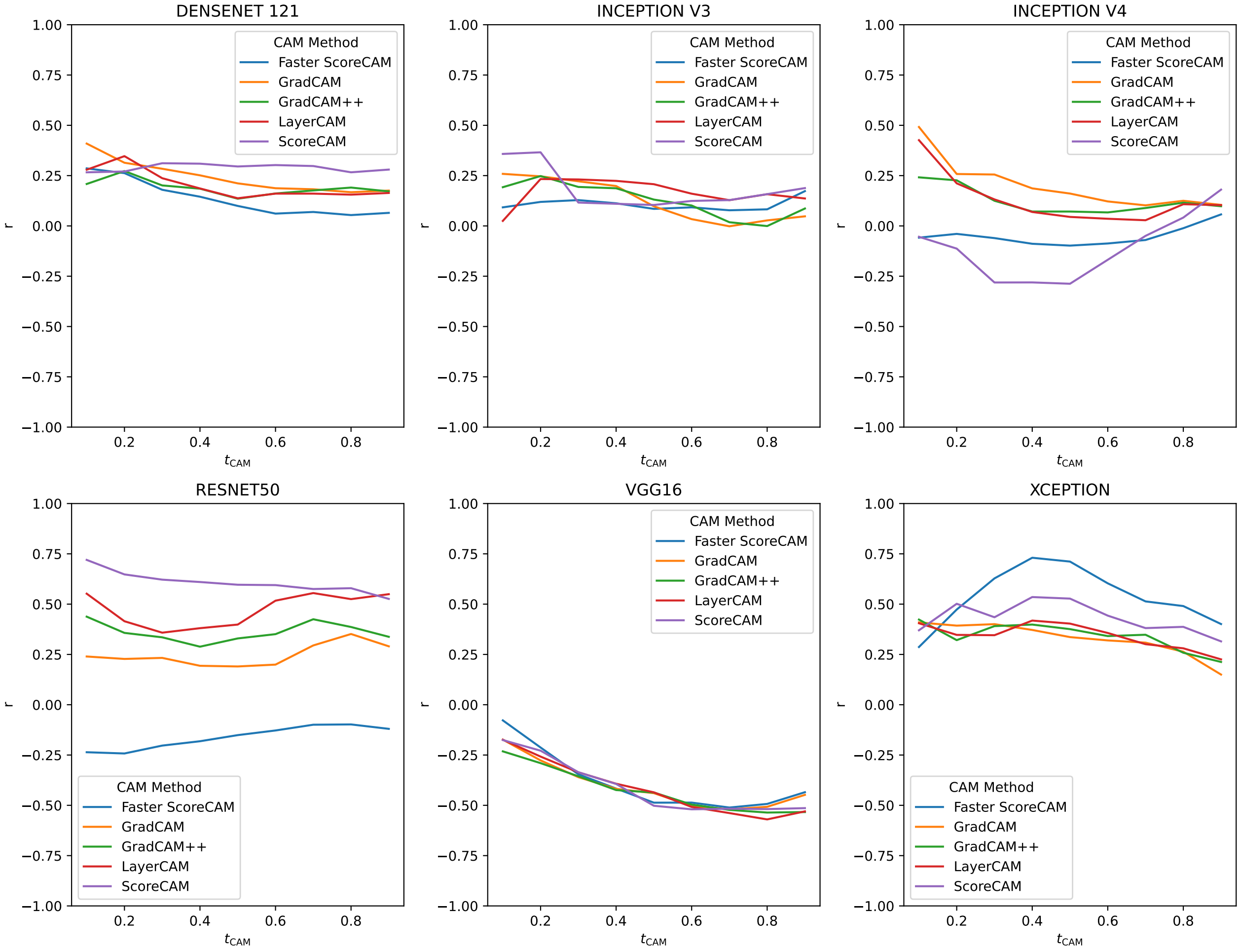

Figure S6: Spearman's rank (r) correlation (y-axis) of damaged CAM regions ($y = 1$) with the amount of liquid loss for each CNN model and for various t_{CAM} threshold values (x-axis).

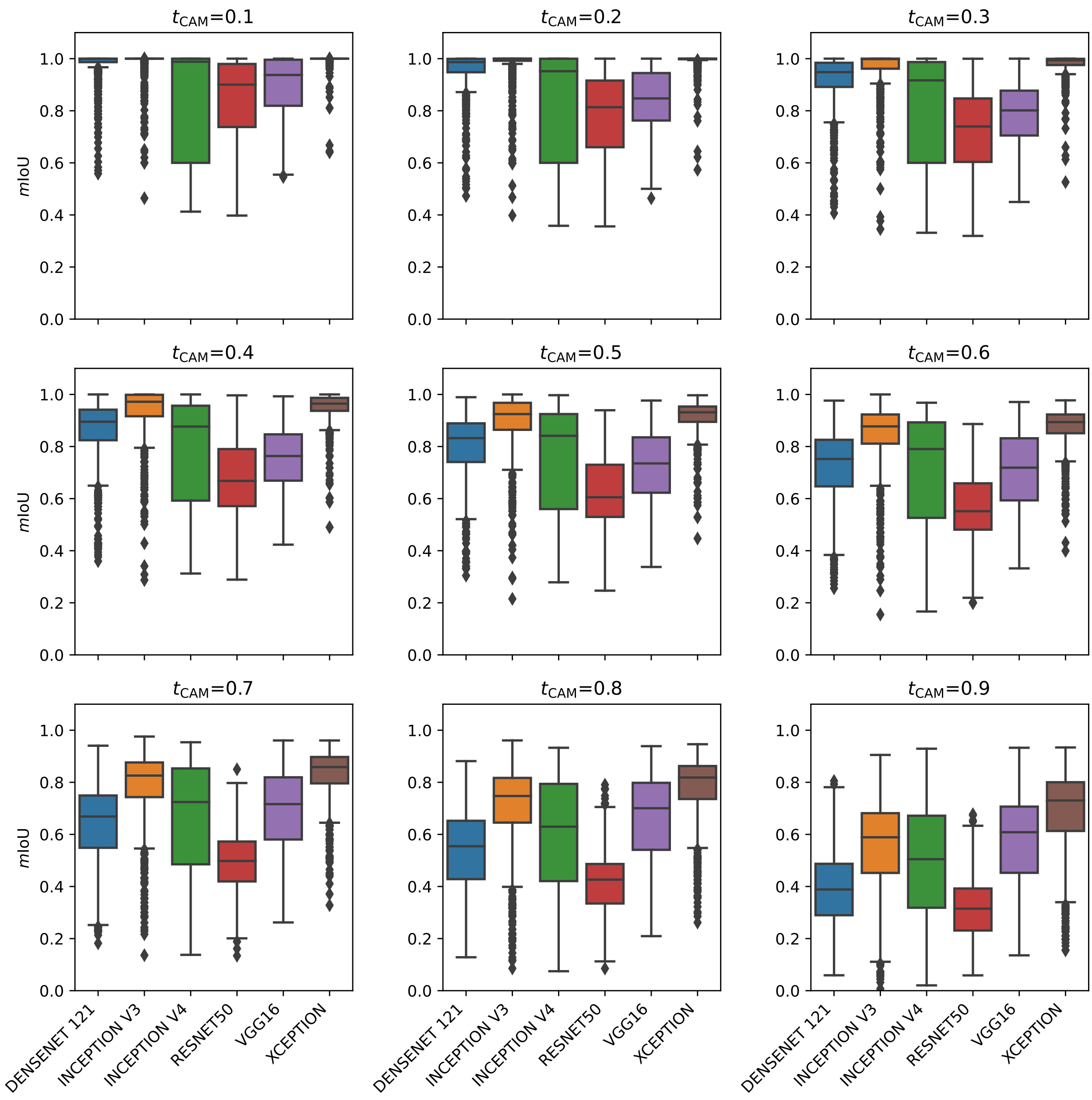

Figure S7: The IoU agreement across CAM methods and for different t_{CAM} threshold values. It reflects the degree to which CAM activations obtained from different CAM methods have a high IoU with each other while fixing the CNN model.

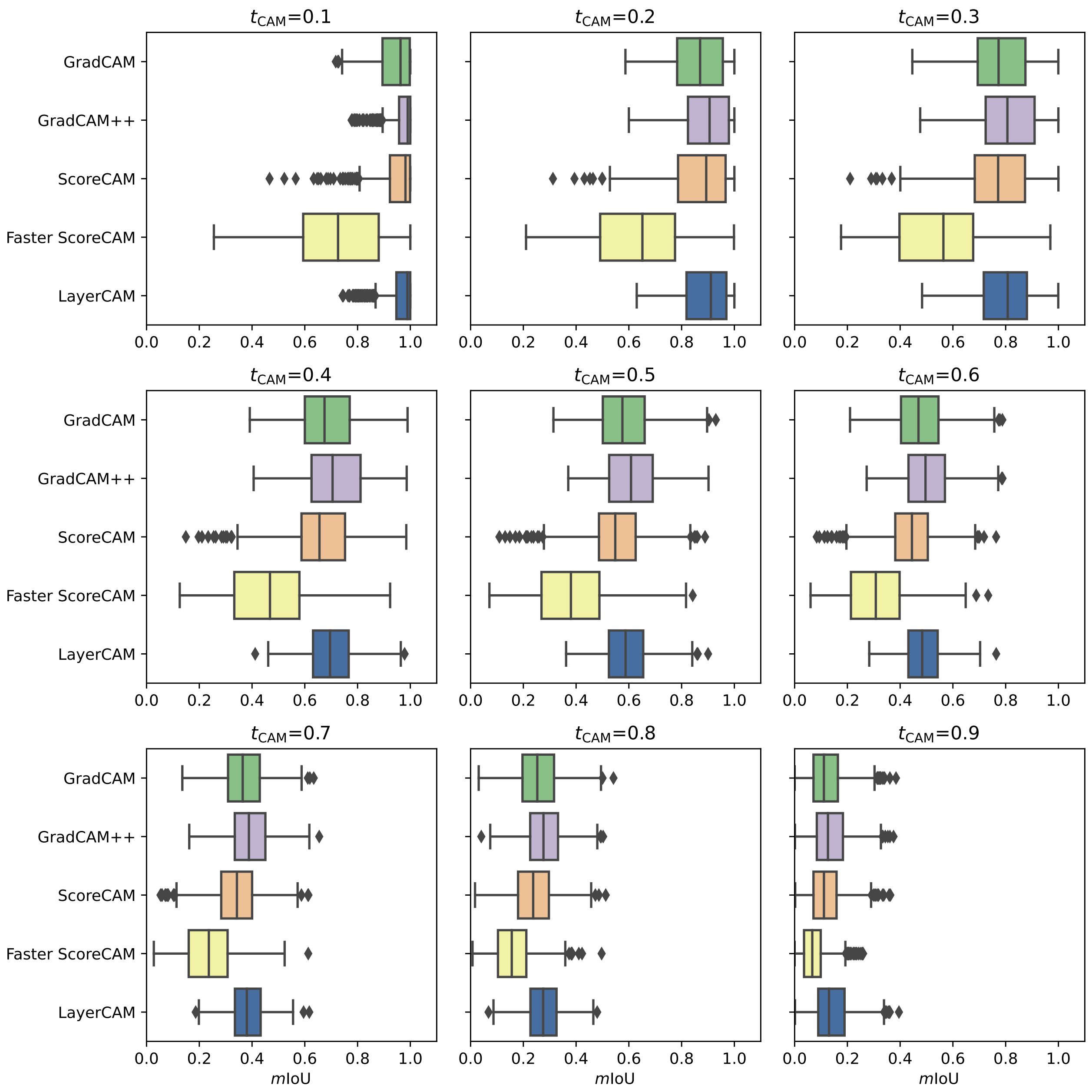

Figure S8: The IoU agreement across different CNN models and for different t_{CAM} threshold values while fixing the CAM method. This agreement is measured by calculating the average intersection over union between unique combinations of CNN models.
